# ATP citrate lyase inhibitor triggers endoplasmic reticulum stress to induce hepatocellular carcinoma cell apoptosis via p‐eIF2α/ATF4/CHOP axis

**DOI:** 10.1111/jcmm.16235

**Published:** 2021-01-03

**Authors:** Yihu Zheng, Qingqing Zhou, Chang Zhao, Junjian Li, Zhengping Yu, Qiandong Zhu

**Affiliations:** ^1^ Departments of Hepatobiliary Surgery The First Affiliated Hospital of Wenzhou Medical University Wenzhou China; ^2^ Departments of Nursing The First Affiliated Hospital of Wenzhou Medical University Wenzhou China

**Keywords:** ACLY inhibitor BMS‐303141, ATP citrate lyase, endoplasmic reticulum stress, hepatocellular carcinoma, sorafenib

## Abstract

ATP citrate lyase (ACLY), a key enzyme in the metabolic reprogramming of many cancers, is widely expressed in various mammalian tissues. This study aimed to evaluate the effects and mechanisms of ACLY and its inhibitor BMS‐303141 on hepatocellular carcinoma (HCC). In this study, ACLY was highly expressed in HCC tissues, especially in HepG2 and Huh7 cells, but was down‐regulated in Hep3B and HCC‐LM3 cells. Besides, ACLY knockdown inhibited HepG2 proliferation and clone formation, while opposite result was noticed in HCC‐LM3 cells with ACLY overexpression. Moreover, ACLY knockdown impeded the migration and invasion abilities of HepG2 cells. Similarly, BMS‐303141 suppressed HepG2 and Huh‐7 cell proliferation. The p‐eIF2α, ATF4, CHOP p‐IRE1α, sXBP1 and p‐PERK were activated in HepG2 cells stimulated by BMS‐303141. In cells where ER stress was induced, ATF4 was involved in BMS‐303141‐mediated cell death procession, and ATF4 knockdown reduced HCC cell apoptosis stimulated by BMS‐303141. In a mouse xenograft model, combined treatment with BMS‐303141 and sorafenib reduced HepG2 tumour volume and weight. In addition, ACLY expression was associated with HCC metastasis and tumour‐node‐metastases staging. Survival analysis and Cox proportional hazards regression model showed that overall survival was lower in HCC patients with high ACLY expression; AFP level, TNM staging, tumour size and ACLY expression level were independent risk factors affecting their overall survival. In conclusion, ACLY might represent a promising target in which BMS‐303141 could induce ER stress and activate p‐eIF2α/ATF4/CHOP axis to promote apoptosis of HCC cells, and synergized with sorafenib to enhance the efficacy of HCC treatment.

## 1 INTRODUCTION

Primary liver cancer is a global refractory malignant tumour with high morbidity and high mortality, and more than 90% of primary liver cancer is hepatocellular carcinoma (HCC).^1^ The occurrence of HCC is associated with chronic inflammation, non‐alcoholic steatohepatitis, alcoholic steatohepatitis and chronic liver injury.^2^ Therefore, in the process of abnormal differentiation of hepatocytes, these pathogenic factors induce endoplasmic reticulum (ER) stress through oxidative stress, inflammation and gene mutations and initiate related cascade reactions.^3^ There are three ER stress sensors including inositol‐requiring 1 (IRE1), activated transcription factor 6 (ATF6) and ER‐resident PKR‐like eIF2α kinase (PERK); these sensors detect the accumulation of unfolded or misfolded protein at the onset of ER stress and initiate the unfolded protein response (UPR).^4^ Besides, the activations of inositol‐requiring protein‐1 α (IRE1α) and protein kinase RNA‐like ER kinase (PERK) can also trigger UPR response. PERK is a major transducer of the ER stress response and directly phosphorylate α‐subunit of eukaryotic initiation factor 2 (eIF2α),^5^ and phosphorylated eIF2α (p‐eIF2α) promotes translation of activating transcription factor 4 (ATF4).^6^ As a pivotal transcription factor in the ER stress pathway, ATF4 mediates the induction of pro‐death transcriptional regulator CCAAT enhancer‐binding protein homologous protein (CHOP).^7^ Previous study has suggested that ER stress and its mediated apoptosis are closely related to the occurrence and development of HCC.^8^

In addition, a recent research has revealed that one of the essential factors in the regulation of tumorigenesis and progression is reprogramming of tumour cell metabolism.^9^ Metabolic reprogramming of tumour cells redefines the flux and flow of nutrients in the metabolic network of tumour cells, so as to meet the specific needs of tumour cell material metabolism and energy metabolism, thereby ensuring the survival and proliferation of tumour cells and maintaining the survival advantage of tumour cells under adverse conditions. Therefore, the analysis of crucial enzymes in reprogramming of tumour cell metabolism will help to understand the mechanism of tumorigenesis and progression, thus finding a promising target for targeted therapy.

ATP citrate lyase (ACLY), a critical enzyme in the metabolic reprogramming of many cancers, is the first rate‐limiting enzyme in the de novo synthesis of fatty acids and is widely expressed in a variety of mammalian tissues.^10^ Citric acid produced by sugar metabolism in cytoplasm can be catalytically cracked by ACLY to oxaloacetate and acetyl‐CoA, which are the basic raw materials for the synthesis of fatty acids and cholesterol; acetyl‐CoA can also be used for protein modification, such as histone acetylation, which is crucial for the transcription and activation of target genes.^11^ It is well documented that ACLY overexpression can maintain the malignant proliferation of various tumour cells and promote their malignant evolution. Furthermore, ACLY is up‐regulated to different degrees in osteosarcoma, renal, cervical, breast, prostate and lung cancers.^12^, ^13^, ^14^ Additionally, Chen et al^15^ have believed the possibility of ACLY as a potential and independent biomarker for the recurrence prediction in breast cancer patients. However, the role and molecular mechanism of ACLY and its inhibitor BMS‐303141 in liver cancer are still unclear.

This study was designed to test the hypothesis that ACLY can promote the proliferation, migration and invasion of HCC, while its inhibitor BMS‐303141 alleviates this effect by triggering ER stress through activation of p‐eIF2α/ATF4/CHOP axis; moreover, ACLY in combination with sorafenib can improve the efficacy of HCC therapy.

## 2 METHODS AND MATERIALS

### 2.1 The Cancer Genome Atlas (TCGA) and Gene Expression Omnibus (GEO) databases

To investigate the ACLY expression profile in HCC and non‐tumour liver tissue samples, we used the RNA sequencing and microarray gene profiling data from GEO (GSE14520) and TCGA. There were 374 existing liver cancer sequencing data in TCGA database (September 2016). Among them, 50 paired liver cancer samples and matched tumour‐adjacent normal samples were included in the differential expression analysis, and 370 complete clinical data of survival data were extracted for follow‐up survival analysis (3 cases with recurrent tumour tissues and 1 case without survival time data were excluded). The sequencing data were obtained from Illumina HiSeq 2000 sequencing platform. GSE14520 dataset was downloaded from Gene Expression Omnibus (GEO) was enrolled in survival analysis.

### 2.2 Patient samples and cell culture

The first group of liver cancer tissue samples were from 105 patients who underwent surgery for liver cancer between January 2007 and December 2009. The second group of liver cancer tissues and adjacent tissues were from 15 patients who underwent hepatectomy for primary liver cancer from May to October 2018. Patients in both groups were from the First Affiliated Hospital of Wenzhou Medical University. Inclusion criteria: (a) patients were confirmed as primary hepatocellular carcinoma by postoperative pathological diagnosis, with complete clinical data; (b) the surgical procedure was radical hepatocellular carcinoma resection, with at least 1‐cm margin far from the tumour tissue; (c) none of the patients had received radiotherapy or chemotherapy before operation; and (d) no distant metastasis was found during the operation. Non‐tumour‐related death was excluded. This study was approved by the Ethics Committee of the First Affiliated Hospital of Wenzhou Medical University (NO.2007‐044), and informed consents were obtained from all patients for the use of their tissue samples.

The human normal liver cell line (LO2) and human HCC cell lines (Hep3B, HepG2, HCC‐LM3, Huh7) were obtained from the cell bank of the Chinese Academy of Sciences (Shanghai, China). All cell lines were routinely incubated in Dulbecco's Modified Eagle Medium (DMEM) with high glucose supplemented with 10% FBS, penicillin G (10 000 U/mL) and streptomycin (10 000 µg/mL) at 37°C in a humidified 5% CO_2_ incubator. When the density of cultured cells reached 80%, they were washed twice in PBS, followed by addition of an appropriate amount of trypsin to digest the adherent cells. Next, the cells were put back into the incubator and let them stand for 3 minutes until the cells showed a discrete state, and then gently tapped the side wall of the culture bottle to make the cells fall off the bottom. Finally, the cells were stopped digesting by adding an appropriate amount of pre‐warmed complete medium.

### 2.3 Real‐time PCR

Total RNA from 15 pairs of HCC tumour tissues and their matched tumour‐adjacent tissues, as well as HCC cells (Hep3B, HepG2, HCC‐LM3, Huh7) and matched tumour‐adjacent cells (LO2), was extracted by TRIZOL Reagent (Invitrogen) following the manufacturer's instructions. Total RNA was pre‐treated with DNase I (Sigma‐Aldrich) and transcribed to cDNA using the Revert Aid First Strand cDNA Synthesis Kit (thermo Fisher Scientific). Real‐time PCR analysis was carried out using a Bestar SYBR Green qPCR Master Mix (DBI Bioscience) on an ABI Prism 7500 on RT‐PCR detector (Applied Biosystems). GAPDH was used as a loading control. All samples were normalized to internal controls, and fold difference was calculated through 2^−ΔΔCt^ method. The primers sequences were list in Table 1.

**TABLE 1:** RT‐PCR primers and siRNA sequences

Primer and siRNA	Sequences
ACLY F	TCGGCCAAGGCAATTTCAGAG
ACLY R	CGAGCATACTTGAACCGATTCT
GAPDH F	GTCTTCACCACCATGGAGAAG
GAPDH R	CAAAGTTGTCATGGATGACCTTGG
siNC sense	UUCUCCGAACGUGUCACGUTT
siNC antisense	ACGUGACACGUUCGGAGAATT
ACLY siRNA1 sense	CCAUCAUAGCUGAAGGCAUTT
ACLY siRNA1 antisense	AUGCCUUCAGCUAUGAUGGTT
ACLY siRNA2 sense	GCACGAAGUCACAAUCUUUTT
ACLY siRNA2 antisense	AAAGAUUGUGACUUCGUGCTT
ATF4 siRNA1 sense	AGGAGCAAAACAAGACAGCATT
ATF4 siRNA1 antisense	AUGCUGUCUUGUUUUGCUCCTT

### 2.4 Western blot

Total protein from 4 pairs of newly operated HCC and normal control tissues, as well as normal liver cell line (LO2) and human HCC cells (Hep3B, HepG2, HCC‐LM3, Huh7), was extracted by RIPA histiocyte rapid lysate (JRDUN, Shanghai, China), supplemented with protease inhibitor of Cocktail (Sigma‐Aldrich). Protein concentrations were measured using BCA protein assay kit (Pierce). Samples containing 20‐30 µg of protein were mixed with SDS sample buffer and boiled for 5 minutes. Proteins were separated by SDS‐polyacrylamide gel electrophoresis using 10% tris‐glycine polyacrylamide gradient gel and transferred to polyvinylidene difluoride membrane (PVDF; Applygen Technologies Inc, Beijing, China). The membranes were blocked with 5% (wt/vol) skimmed milk powder in TBS for 1 hour at room temperature and then incubated with the primary antibodies against ACLY, p‐eIF2α, eIF2α, ATF4, CHOP, PERK, phosphorylated PERK (p‐PERK), IRE1α, phosphorylated IRE1α (p‐IRE1α) and splices X‐box binding protein (sXBP1) (Affinity Biosciences) and GAPDH (Cell Signaling Technology) overnight at 4°C. After 3 washes in TBST, the membrane was then incubated with HRP‐conjugated secondary antibody. Next, the bands were visualized through Gel Doc™ EZ Imager (Bio‐Rad Laboratories). Finally, the density of each target protein in the Western blot was normalized according to the GAPDH loading control to get semi‐quantification of the target protein in each lane.

### 2.5 Overexpression or knockdown system

The full‐length cDNA of ACLY was cloned from human HepG2 cells by RT‐PCR and cloned into mammalian expression vector GV230‐EGFP (GENE, Shanghai, China) to construct overexpression plasmids, and the empty vector (vector) was used as a negative control. The two siRNAs of ACLY (siRNA1‐2) and nonsense RNAi (si‐NC) were designed and synthesized by Shanghai Biotech. The primers and siRNA sequences were list in Table 1. Firstly, HCC‐LM3 and HepG2 cells were inoculated into a 6‐well plate, respectively. Then, 5 μL of liposome transfection reagent Lipofectamine 2000 (Invitrogen) and 250 μL of DMEM medium were previously mixed into DMEM medium with siRNA or 4 μg of plasmid according to the transfection instructions. Transfection was done when the cells adhered to the bottom of the plates, and the cell density in the cell plate was observed to be about 60%‐70%. Then, a mixture of siRNA‐lipo2000‐DMEM and GV230‐EGFP‐ACLY‐lipo2000‐DMEM was separately added to the prepared HepG2 and HCC‐LM3 cells. Transfection efficiency of PARP‐1 siRNA or PARP‐1 plasmid was detected by Western blot.

### 2.6 Cell proliferation assay

The effect of ACLY on the proliferation of HepG2 and HCC‐LM3 cells was detected by Cell Counting Kit‐8 (CCK‐8) assay. Firstly, HepG2 and HCC‐LM3 cells were seeded into 96‐well plates at a density of 2 × 10^3^ cells per well with one group of untreated (control), treated with vehicular control (HepG2‐siNC) and siRNA2 of ACLY (HepG2‐siRNA2), and another group of untreated (control), treated with vehicular control (HCC‐LM3‐vector) and ACLY expression plasmids (HCC‐LM3‐ACLY). The cells continued to culture for 24, 48, 72, 96 and 120 hours after transfection, then 100 μL Cell Counting Kit‐8 (CCK‐8) solution was added per well, and plates were incubated at 37°C for 1 hour. The absorbance was measured at 595 nm using a microplate reader.

### 2.7 Cell viability assay

After administration of ACLY inhibitor BMS‐303141, HepG2 and Huh‐7 cells was treated with MTT dye [3‐(4,5‐dimethylthiazol‐ 2‐yl)‐2,5‐diphenyltetrazolium bromide] at 37°C for 3 hour. The formazan crystals were dissolved in DMSO, and absorbance was measured at a wavelength of 540 nm in a microplate reader. The concentration of drug to inhibit 50% of cell viability (IC50 value) was calculated using CalcuSyn software.

### 2.8 Colony formation assay

Colony formation assay was adopted to evaluate the effects of ACLY on HepG2, HCC‐LM3 and Huh‐7 cells’ proliferation and tumorigenicity in vitro. Cells were plated in a 6‐well plate containing 1 × 10^4^ cells per well in triplicate and divided into two groups. After 2 weeks, the cells were fixed with 4% paraformaldehyde for 15 minutes and stained with 0.1% crystal violet. The visible colonies were counted.

### 2.9 Transwell migration and invasion assays

Transwell assays were performed to examine the effects of ACLY on cell migration and invasion. Cells in serum‐free medium were seeded into the upper chamber in triplicate. The transwell filter chambers were pre‐coated with or without Matrigel (BD Biosciences), and the cell medium was supplemented with 10% FBS placed in the lower chamber. After 36‐48 hours of incubation, migrated cells were fixed in 4% paraformaldehyde, stained with 0.1% crystal violet, imaged and counted.

### 2.10 FITC‐annexin V/PI double staining

HepG2 cells were pre‐treated with vehicular control or siRNA specific for ATF4 (siRNA1), and then seeded in 6‐well plates. After the cells adhered to the bottom of the plates, they were either stimulated with or without BMS‐303141. An Annexin V‐FITC/PI (fluorescein isothiocyanate/propidium iodide) double‐staining apoptosis detection kit (BD Pharmingen; San Diego, CA, USA) was used to detect apoptotic cells via flow cytometry (Becton, Dickinson and Company, USA) following the manufacturer's protocols. A minimum of 3 × 10^4^ cells were analysed for each sample. All experiments were repeated 3 times.

### 2.11 Tumour xenograft model

Totally, 16 BALB/c nude mice (4‐5 weeks old) were purchased from Shanghai Slac Laboratory Animal Corporation. HepG2 cells (5 × 10^7^ cells/mouse) were injected into the abdominal subcutaneous of the forelimb of mice. When the tumour volume reached 100 mm^3^, mice were randomly assigned into 4 groups (n = 4 per group) and given suspensions containing the following components: normal saline, BMS‐303141, sorafenib, and a combination of BMS‐303141 and sorafenib by gavage at a dosage of 5 mg/kg/d for 8 days. After treatment for 8 days, tumour volumes (V) were measured every 2 days using the formula: V (mm^3^) = 0.5 × length × width^2^. In addition, the bodyweights of the mice were simultaneously recorded for accurately reflecting changes in the tumour. At the end of the experiment, the bodyweights of the mice were first measured, then the mice were killed, and the tumours in nude mice were taken out, measured with a vernier caliper, weighed and photographed. In addition, the proliferation of tumour was determined by Ki‐67 immunostaining in randomly selected fields and imaged. Protocols involving the use of the animals were approved by the First Affiliated Hospital of Wenzhou Medical University.

### 2.12 Statistical analysis

Experimental statistical analysis was performed using SPSS 23.0 statistical software. ANOVA analysis was applied to assess interactions between groups and differences between means. The cumulative survival rate of survival data was calculated by Kaplan‐Meier. Log‐rank was used to compare the survival rate between groups. Cox regression analysis was stepwise regression method. A *P* value of <.05 was considered to be statistically significant.

## 3 RESULTS

### 3.1 ACLY is highly expressed in HCC samples

**FIGURE 1:**
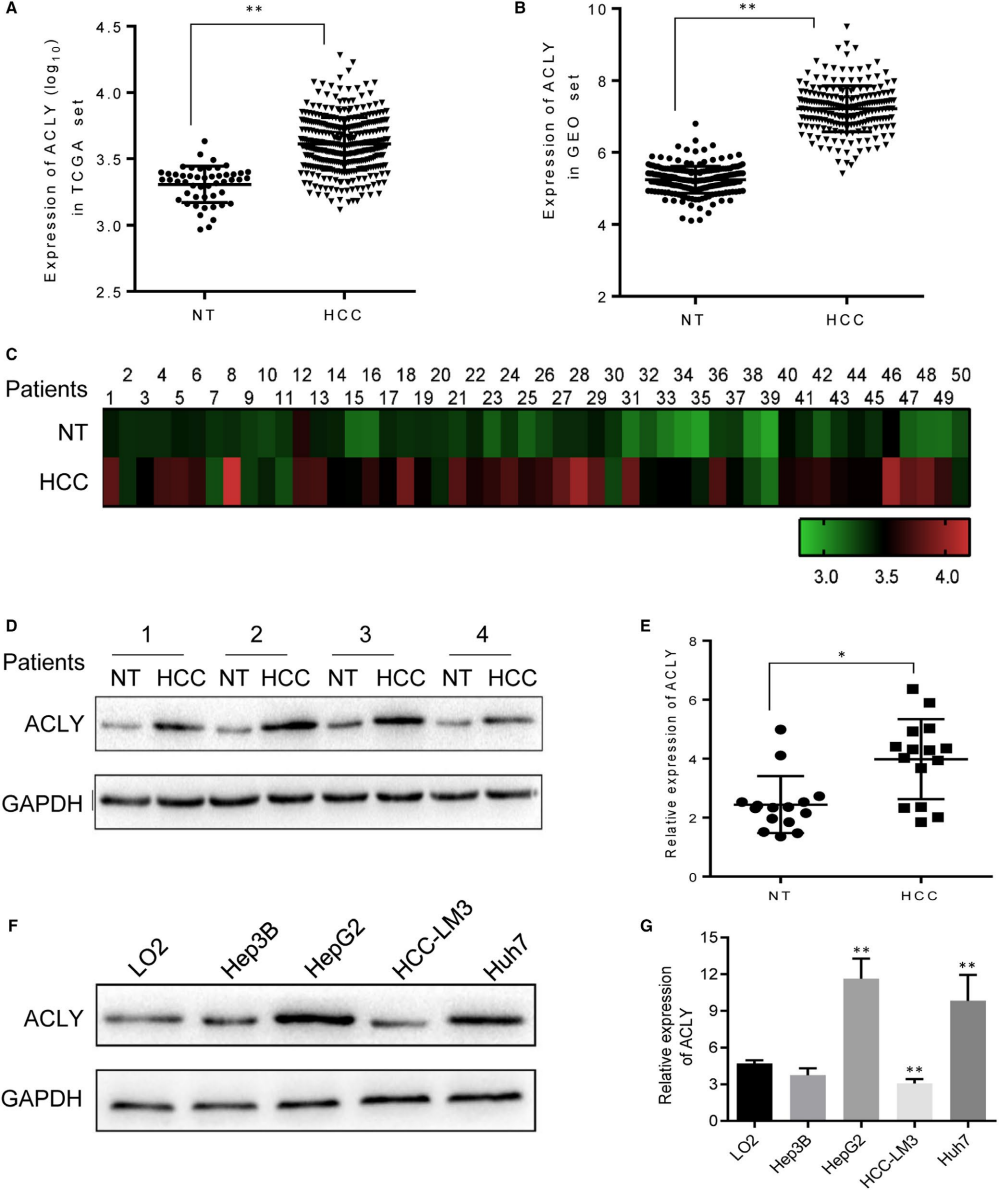
ACLY is highly expressed in HCC. RNA sequencing and microarray gene profiling data from (A) TCGA and (B) GEO was utilized to investigate ACLY expression in HCC samples (n = 370) and non‐tumour liver tissue samples (n = 50). C, We also took HCC samples (n = 50) and normal liver samples (n = 50) from TCGA data to assess ACLY expression. D, Western blot was performed to determine the protein expression of ACLY in newly operated HCC (n = 4) and adjacent normal tissue specimens (n = 4) from patients. E, The mRNA level of ACLY was detected in HCC tissues (n = 15) compared with that in the adjacent normal tissues (n = 15) by real‐time PCR. (^*^ *P* < .05, ^**^ *P* < .01, compared with NT group) (F) Western blot and (G) Real‐time PCR were adopted to detect the protein and mRNA expressions of ACLY in human normal liver cell line (LO2) and human HCC cell lines (Hep3B, HepG2, HCC‐LM3, Huh7), respectively. (^**^ *P* < .01, compared with LO2 group). All experiments were repeated 3 times

According to TCGA and GEO databases, ACLY was found to exhibit significantly higher expression levels in HCC tissues than that in normal liver tissues (both *P* < .01) (Figure 1A‐B). We also took 50 HCC samples and 50 normal liver samples from TCGA data, and the ACLY expression was highly expressed in HCC tissues (Figure 1C). To further determined the expression of ACLY in HCC, we selected 4 pairs of newly operated HCC and adjacent normal tissue specimens from patients. As shown in Figure 1D, ACLY was up‐regulated in HCC tissues compared with that in the adjacent normal tissues. In addition, the mRNA level of ACLY was increased in 15 HCC tissues as shown by RT‐PCR (Figure 1E). Subsequently, to figure out the relationship between ACLY expression and human HCC cell lines, Western blot analysis was performed. Results showed that the mRNA and protein expression of ACLY was remarkably higher in HepG2 and Huh7 cells, but lower in Hep3B and HCC‐LM3 cells (Figure 1F‐G). Therefore, HepG2 was selected as the high‐expression ACLY cell line and HCC‐LM3 as the low‐expression cell line for subsequent experiments.

### 3.2 Up‐regulated ACLY expression is strongly related with disease progression

The correlations between ACLY expression and clinicopathologic features of HCC were further analysed. Hepatocellular carcinoma samples were divided into low‐expression group and high‐expression group according to expression grade of ACLY. Grade 0 and I were in the low‐expression group, while grade II and III were in the high‐expression group. The results showed that the expression of ACLY was significantly associated with HCC metastasis (*P* = .025), but not gender, age, hepatitis B (HBV) viral status, tumour‐node‐metastases (TNM) staging, alpha‐fetoprotein (AFP), cirrhosis, main tumour size, multimode and portal vein tumour thrombus (PVTT) (Table 2).

**TABLE 2:** Clinicopathological data

Characteristics	ACLY (number)		
	High expression	Low expression	*P* value
Gender			.324
Male	51	42	
Female	8	4	
Age (y)			.265
<50	26	24	
>=50	33	22	
HBV viral status			.110
N	13	16	
Y	46	30	
AFP (ng/ml)			.505
<400	31	25	
>=400	28	21	
Cirrhosis			.137
N	3	6	
Y	56	40	
TNM staging			.076
I	15	20	
II	30	14	
III	14	12	
Main tumour size (cm)			.464
>5	35	26	
<=5	24	20	
Multi‐node			.129
N	50	34	
Y	9	12	
PVTT			.500
N	41	32	
Y	17	12	
Metastasis			.025
N	46	43	
Y	13	3	

Abbreviations: AFP, alpha‐fetoprotein; HBV viral status, hepatitis B viral status; PVTT, portal vein tumour thrombus; TNM staging, tumour‐node‐metastases staging.

**FIGURE 2:**
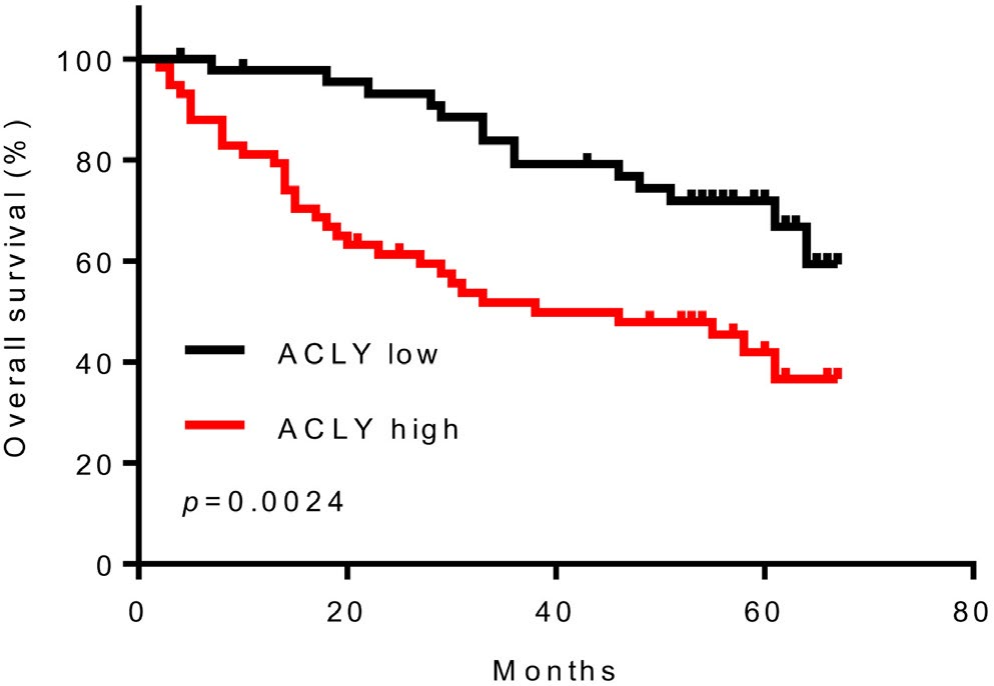
HCC patients with high ACLY expression had shorter overall survival rate. Overall survival rate of HCC patients with high or low ACLY expression levels (n = 105). This experiment was repeated 3 times

Then, the classification of ACLY expression was included in multivariate analysis and established Cox proportional hazards regression model. The results showed that AFP level, TNM staging, tumour size and ACLY expression level were independent risk factors affecting the overall survival rate of HCC patients (Table 3). In addition, stratification of ACLY mRNA showed a strong association between ACLY and overall survival rate of patients with HCC. As shown in Figure 2, patients with high ACLY expression had shorter overall survival rate than patients with low ACLY expression.

**TABLE 3:** Cox regression analysis of overall survival rate of HCC patients

Variables	*P* value	Hazard ratio	95% CI	
			Lower limit	Upper limit
Gender	.163	0.392	0.105	1.460
Age	.511	1.242	0.652	2.365
HBV viral status	.169	1.802	0.779	4.170
AFP	.008	0.368	0.176	0.772
Cirrhosis	.239	0.422	0.100	1.774
TNM staging	.007	2.341	1.265	4.334
Main tumour	.048	0.496	0.248	0.992
Multi‐node	.683	0.825	0.327	2.078
PVTT	.485	1.323	0.603	2.902
Metastasis	.110	0.481	0.196	1.180
ACLY	.000	4.512	2.045	9.954

Abbreviations: 95% CI, 95% confidence intervals; ACLY, ATP citrate lyase; AFP, alpha‐fetoprotein; HBV viral status, hepatitis B viral status; PVTT, portal vein tumour thrombus; TNM staging, tumour‐node‐metastases staging.

### 3.3 ACLY promotes proliferation, migration and invasion of HCC cells

**FIGURE 3:**
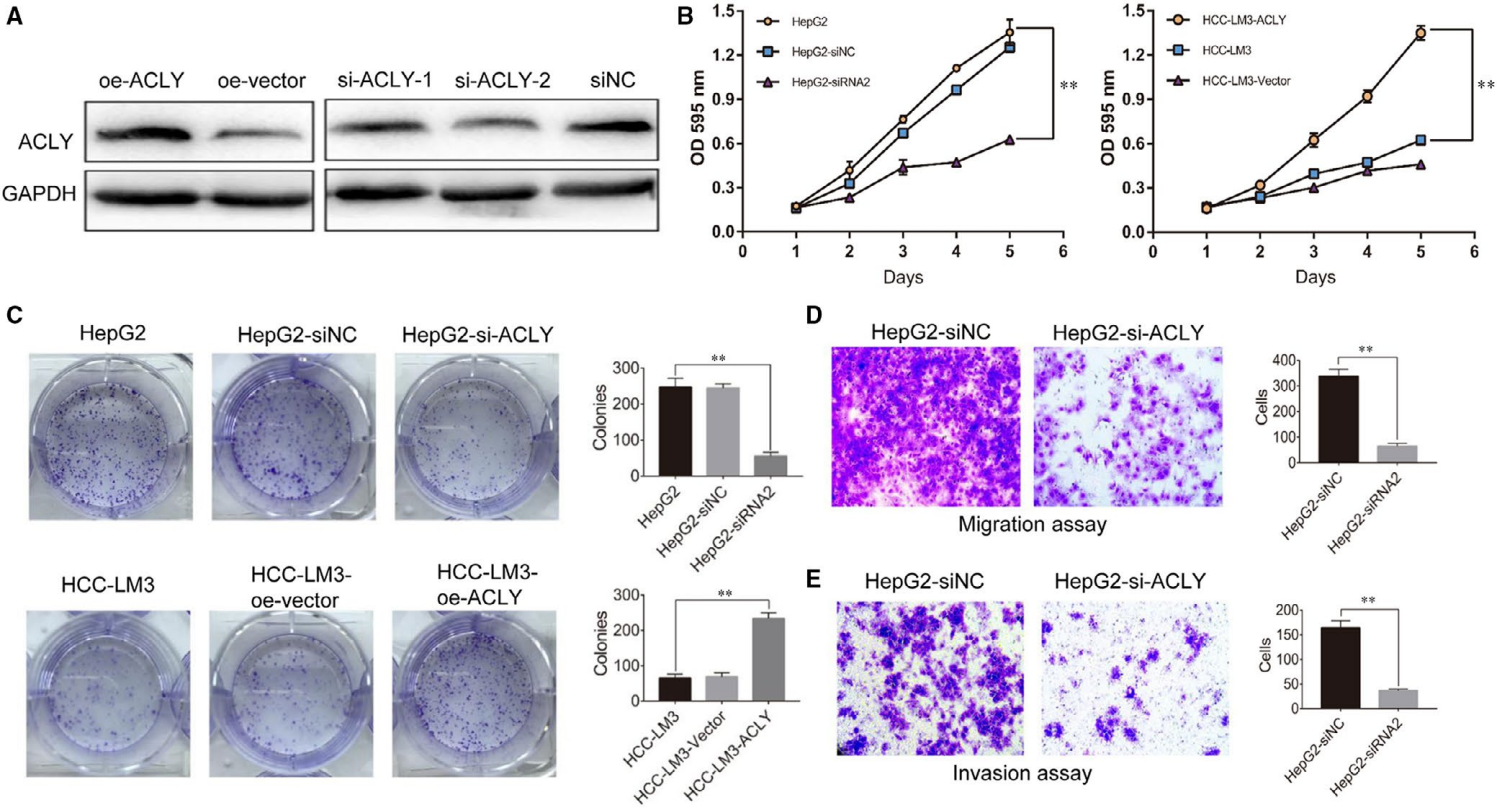
ACLY promotes HCC cells proliferation, migration and invasion. We constructed HCC‐LM3 cells with ACLY overexpression, and HepG2 cells with ACLY knockdown. (A) Western blot was utilized to investigate ACLY expression in HCC‐LM3 and HepG2 cells after construction. The effects of knockdown or overexpression of ACLY on proliferation and clone formation of HepG2 and HCC‐LM3 cells were evaluated using (B) CCK‐8 assay and (C) colony formation assay. (^**^ *P* < .01, compared with HepG2 group or HCC‐LM3 group) (D) Cell migration and(E) invasion assays were performed to test the hypothesis that the overexpression of ACLY may be related to the invasion and migration of HCC cells. (^**^ *P* < .01, compared with HepG2‐siNC group). All experiments were repeated 3 times

To investigate the biological function of ACLY in pathogenesis of HCC, we constructed HCC‐LM3 cells with ACLY overexpression and HepG2 cells with ACLY knockdown using siRNAs of ACLY (siRNA‐1 and siRNA‐2). As shown in Figure 3A, lentivirus‐mediated overexpression or knockdown systems were established and noticed that siRNA‐2 had a better effect in knocking down ACLY than siRNA‐1. Therefore, siRNA‐2 was used in subsequent experiments. CCK‐8 assay and colony formation results indicated that ACLY overexpression significantly promoted the proliferation and colony number of HCC‐LM3 cells, while the opposite result was noticed from HepG2 cells with ACLY knockdown (Figure 3B,C). Furthermore, it is well documented that the expression of ACLY was significantly correlated with TNM staging of HCC in this study. Then, cell migration and invasion assay were performed to test the hypothesis that the overexpression of ACLY may be related to the invasion and migration of HCC cells. As expected, ACLY knockdown significantly suppressed the migration and invasion ability of HepG2 cells (Figure 3D,E).

### 3.4 ACLY inhibitor suppresses HCC cell viability and colony number

**FIGURE 4:**
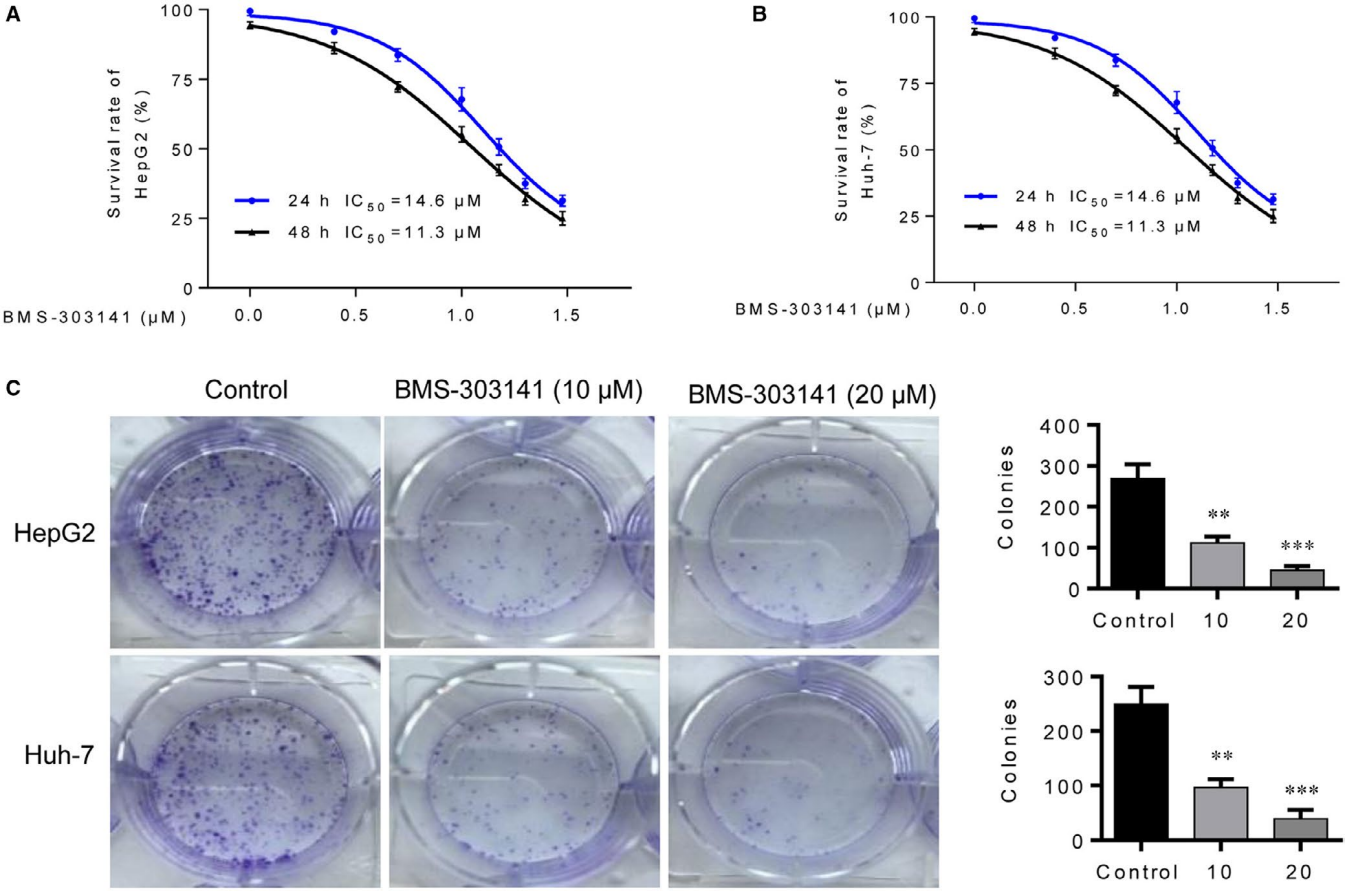
ACLY inhibitor suppresses HCC cell viability and colony number. MTT assay was utilized to assess (A) HepG2 and (B) Huh‐7 cell viability after administration of ACLY inhibitor BMS‐303141. C, The effects BMS‐303141 on clone formation of HepG2 and Huh‐7 3 cells were evaluated using colony formation assay. (^**^ *P* < .01, ^***^ *P* < .001, compared with control group). All experiments were repeated 3 times

To identify the effect of BMS‐303141 on the proliferation of HepG2 and Huh7 cells with different up‐regulation of ACLY, MTT and colony formation assays were performed. From Figure 4, stimulation of BMS‐303141 at 10 μmol/L or 20 μmol/L significantly suppressed the proliferation and colony number of HepG2 and Huh7 cells.

### 3.5 ACLY inhibitor triggers ER stress and activate p‐eIF2α/ATF4/CHOP axis in vitro

**FIGURE 5:**
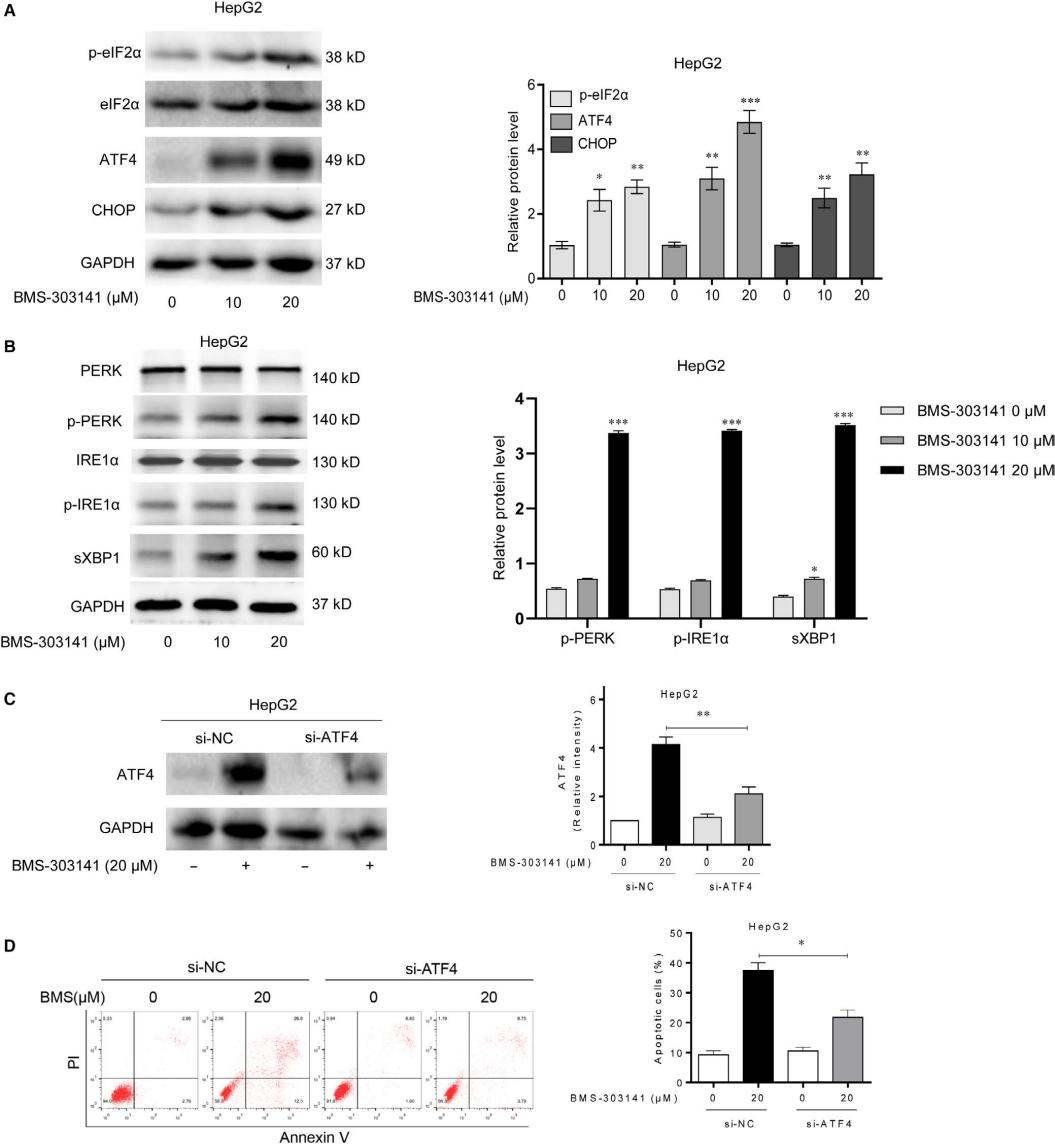
ACLY inhibitor triggers ER stress and activates p‐eIF2α/ATF4/CHOP axis in vitro. Western blot analysis of (A) ER stress‐related proteins (p‐eIF2α, eIF2α, ATF4 and CHOP) and (B) UPR signal transduction molecules (p‐PERK, PERK, p‐IRE1α, IRE1α and sXBP1) in HepG2 cells after administration of BMS‐303141. ATF4p‐eIF2α, eIF2α were activated 3 h post‐treatment; CHOP was activated 8 h post‐treatment. (^*^ *P* < .05, ^**^ *P* < .01 and ^***^ *P* < .001, compared with control group) (C) Western blot analysis of protein expression after ATF4 knockdown. (D) Annexin V‐FITC/PI double staining was performed to determine the apoptosis rate of HepG2 cells after ATF4 knockdown via flow cytometry. (^*^ *P* < .05, ^**^ *P* < .01 and ^***^ *P* < .001, compared with con siRNA group). All experiments were repeated 3 times

Unfolded protein response is an important cellular that responses to ER stress. To find out whether ER stress can be triggered after BMS‐303141 exposure, ER stress‐related proteins (eIF2α, p‐eIF2α, ATF4 and CHOP) and UPR signal transduction molecules (p‐PERK, PERK, p‐IRE1α, IRE1α and sXBP1) were measured via Western blot. As shown in Figure 5A, p‐eIF2α, ATF4 and CHOP were obviously elevated in a dose‐dependent manner after BMS‐303141 treatment, while no significant change was observed in eIF2α. Additionally, BMS‐303141 induced up‐regulated protein expression of sXBP‐1, p‐IRE1α and p‐PERK when compared with control cells, suggesting that BMS‐303141 activated UPR, which might probably due to ER stress (Figure 5B).

Considering the key role of ATF4 in cell apoptosis, siRNA1 was used to knock down ATF4 expression. The transfection of si‐ATF4 reduced the ATF4 expression in HepG2 cells compared with control group (Figure 5C). Subsequently, Annexin V‐FITC/PI double‐staining and flow cytometry assay was used to evaluate the apoptotic rate of HepG2 cells after lentivirus‐mediated ATF4 knockdown. As expected, the apoptosis of ATF4 knockdown cells was notably reduced after BMS‐303141 administration (Figure 5D). These findings indicated that BMS‐303141 triggered ER stress and induced HepG2 cell apoptosis via the activation of p‐eIF2α/ATF4/CHOP axis.

### 3.6 ACLY inhibitor amplifies the therapeutic effect of sorafenib in the treatment of HCC in vivo

**FIGURE 6:**
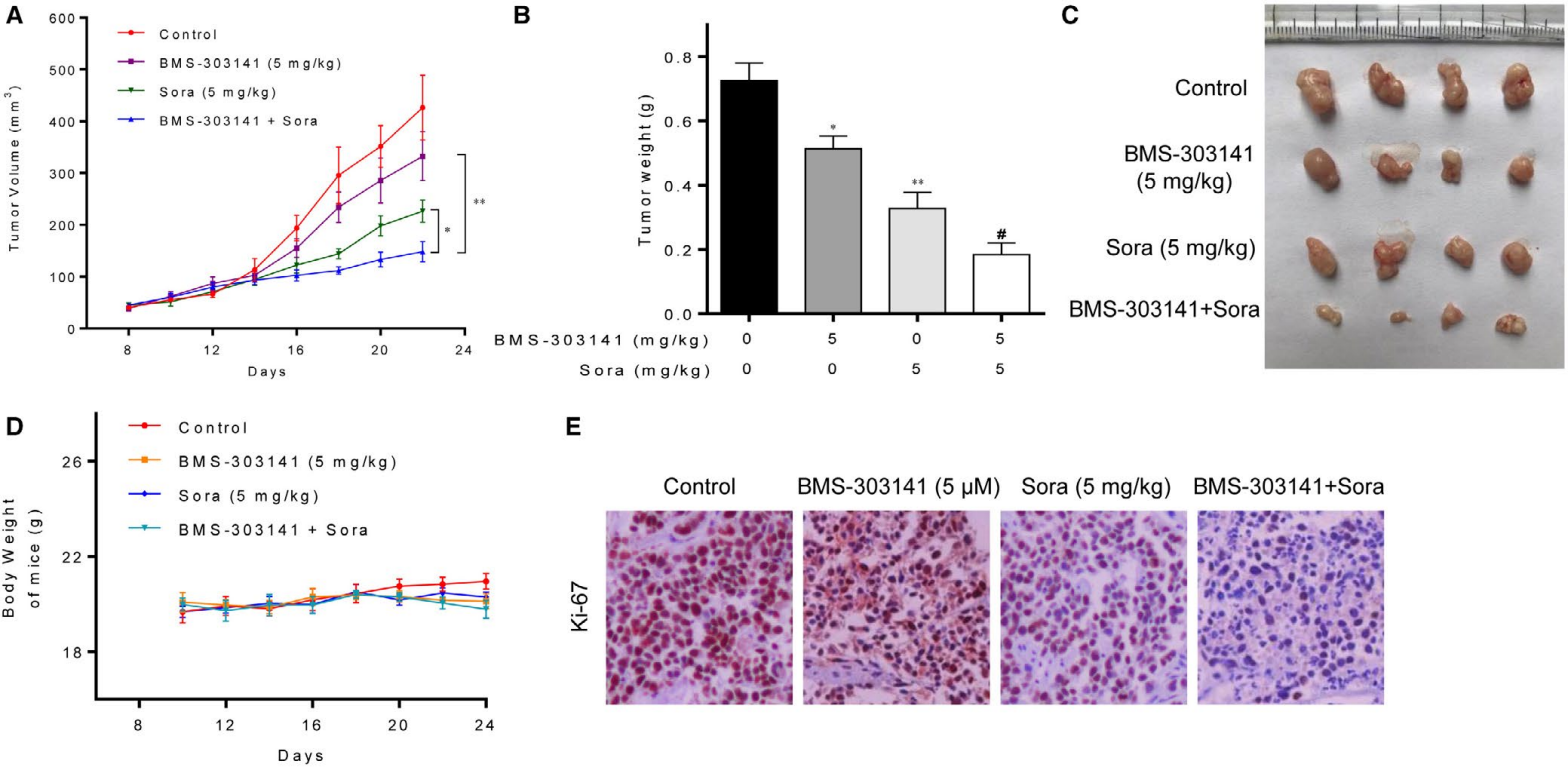
ACLY inhibitor amplifies the therapeutic effect of sorafenib in the treatment of HCC in vivo. Totally, 16 BALB/c nude mice were divided into 4 groups (n = 4 per group) and given suspensions containing the following components: normal saline, BMS‐303141, sorafenib, and a combination of BMS‐303141 and sorafenib by gavage at a dosage of 5 mg/kg/d for 8 d. HepG2 (A) tumour volume, (B) tumour weight and (C) tumour morphology in athymic nu/nu mice after treatment with sorafenib and BMS‐303141. (D) The weight of mice changed at different times after administration with sorafenib and BMS‐303141. (E) Ki‐67 staining of HepG2 tumour cells after treatment with sorafenib and BMS‐303141. (^*^ *P* < .05, ^**^ *P* < .01 and ^***^ *P* < .001, compared with control group; ^#^ *P* < .05, compared with sorafenib alone group). All experiments were repeated 3 times

To evaluate the synergetic effect of sorafenib and BMS‐303141 in vivo, we injected HepG2 cells in athymic nu/nu mice. When the tumours grew to about 100 mm^3^, mice were treated with indicated compounds. As shown in Figure 6A‐C, treatment of sorafenib alone inhibited HCC cells growth in mice (*P* < .01). Moreover, combined treatment with BMS‐303141 and sorafenib markedly reduced HepG2 tumour volume and weight compared to sorafenib alone group (*P* < .05). Notably, there was no significant difference of bodyweight in mice among the control and combined treatment groups (Figure 6D). Moreover, Ki‐67 immunostaining result showed that combined treatment with BMS‐303141 and sorafenib remarkably inhibited Ki‐67 expression (Figure 6E). These findings indicated that BMS‐303141 and sorafenib could synergistically reduce HCC cells of HepG2 tumour in vivo.

## 4 DISCUSSION

The most notable finding of this study was that ACLY could enhance the proliferation, invasion and migration of HCC cells, while ACLY inhibitor BMS‐303141 triggered ER stress to induce apoptosis of HCC cells via activation of p‐eIF2α/ATF4/CHOP axis. This finding fills the gap in the impact of ACLY and BMS‐303141 on hepatocellular carcinoma. We first analysed the expression of ACLY in the TCGA and GEO database, and found that ACLY expression in HCC tissues was significantly higher than that in adjacent tissues. In addition, this trend was further testified in clinical pathology specimens by using Western blot and RT‐PCR. Consistently, several researches have suggested ACLY is significantly up‐regulated in a variety of tumours, promoting tumour progression and metastasis.^16^, ^17^ To clarify the significance of ACLY overexpression in HCC tissues, we carried out cytofunctional studies. Results showed that ACLY expression in multiple cell lines was slightly different, among which ACLY expression was relatively highest in HepG2 cell line, while relatively low in HCC‐LM3 cell line, indicating that ACLY expression was different in HCC cells with different malignant degrees.

Up‐regulated ACLY expression in HCC cells revealed that ACLY inhibition or knockdown may be a potential therapy for HCC. Previous studies have showed that ACLY inhibitor (SB‐204990) or ACLY knockdown restrains tumour cell proliferation and survival in a variety of tumours.^18^, ^19^ Georgia et al^20^ have noticed that knockdown of ACLY expression in HCC cells can not only perform the above functions, but also induce differentiation of tumour cells and form glandular structures in tissues. Moreover, ACLY overexpression in colorectal cancer can induce irinotecan resistance, which was reversed by ACLY knockdown.^21^ In the current study, we constructed HCC‐LM3 cells with ACLY overexpression and HepG2 cells with ACLY knockdown, and found that ACLY knockdown significantly impeded the proliferation, migration and invasion of HepG2 cells, while ACLY overexpression promoted HCC‐LM3 proliferation. This confirmed that ACLY had a similar effect to the original oncogene in the progression of HCC, and ACLY knockdown can suppress the proliferation and metastasis of HCC cells.

Apoptosis can be induced when the UPR fails to compete with severe and persistent external stimuli, resulting in ER stress.^22^ Maurel et al^23^ have clarified that ER stress is associated with the pathogenesis of viral hepatitis, hepatic osteogenesis that enhance the risk of HCC. Under severe and prolonged ER stress, ER stress sensors induce cell death by activating downstream molecules.^24^ One of the main pathways of ER stress‐induced apoptosis was PERK/eIF2‐mediated activation of CHOP.^25^ Nevertheless, the underlying mechanism of ER stress during the treatment of ACLY inhibitor BMS‐303141 in HCC remains unclear. We assumed that BMS‐303141 regulates cell apoptosis likely by modulating ER stress and UPR. Guo et al^26^ have pointed out that PERK can induce CHOP expression by phosphorylating eIF2α and activating ATF4. Our data showed that p‐eIF2α, ATF4, CHOP and p‐PERK were rapidly activated in HepG2 cells by BMS‐303141 treatment. It has been shown that IRE1α/XBP1 pathway plays a vital part in cell survival and cell death via up‐regulation of UPR‐related genes.^25^ IRE1 dimerizes and initiates splicing of XBP1 mRNA which is a key regulator of UPR or ER stress response.^27^ Consistent with this result, we also found that BMS‐303141 led to an enhancement in sXBP1 and p‐IRE1α, indicating the activation of UPR. Activating transcription factor 4 is a vital transcription factor involved in ER stress and apoptotic signalling.^28^ To further determine whether the apoptosis induced by BMS‐303141 depended on the expression of ATF4, siRNA1 was used to knock down ATF4 expression. Results clarified that the apoptotic rate of HepG2 cells that treated by BMS‐303141 was decreased after lentivirus‐mediated ATF4 knockdown. These findings demonstrated that BMS‐303141 induced ER stress and activated p‐eIF2α/ATF4/CHOP axis.

Another important finding of this study was that BMS‐303141 potentiated the efficacy of anticancer drug sorafenib in the treatment of HCC. To our best knowledge, sorafenib is a multi‐target kinase inhibitor that widely used in clinical practice of HCC therapy.^29^ Additionally, Youssef et al^30^ have introduced a novel combination therapy for HCC and found that combining sorafenib and Biochanin‐A synergistically promoted the anti‐proliferation and apoptosis of HCC cells. In this study, thymic‐free nu/nu mice were injected with HepG2 cells, and then treated with sorafenib and BMS‐303141 alone or in combination. We found that combined treatment with BMS‐303141 and sorafenib notably reduced HepG2 tumour volume and weight in comparison with sorafenib alone group, demonstrating that BMS‐303141 could synergically enhance sorafenib effect on HCC cancer growth and inhibition in vivo.

Phosphorylated ACLY expression in human lung adenocarcinoma is implicated with stage, differentiation and poor prognosis.^31^ Thus, ACLY overexpression was found to be a significant negative prognostic factor for this type of cancer. To clarify the effect of ACLY expression in HCC tissues on the prognosis of HCC patients, we evaluated 105 HCC tissue specimens. Results suggested that ACLY expression was associated with HCC metastasis. Cox proportional hazards regression analysis indicated that AFP level, TNM staging, tumour size and ACLY expression level were independent risk factors affecting the overall survival rate of HCC patients. Besides, the overall survival rate was significantly higher in patients with low ACLY expression than that with high ACLY expression. AFP is a glycoprotein, which is mainly synthesized in the foetal liver and reaches its peak in the third month of gestation, and then gradually decline.^32^, ^33^ However, HCC resumes the function of producing AFP when liver cells become cancerous, which is a good indicator of HCC screening and postoperative follow‐up.^34^ In our study, AFP and ACLY expression abundance were both independent risk factors affecting the overall survival of patients, but AFP was not associated with ACLY expression, which requires further study in the future.

## 5 CONCLUSION

In conclusion, the present study reveal that ACLY may represent a promising target in which its inhibition BMS‐303141 can induce ER stress and activate p‐eIF2α/ATF4/CHOP axis to promote apoptosis of HCC cells, and enhance the efficacy of HCC treatment through synergy with sorafenib. However, there are several limitations in this study, such as the lack of the assessment of ER stress in HCC cell lines with high and low expression of ACLY, and the evaluation of lipotoxic stress, which require further in‐depth research in the future.

## CONFLICT OF INTEREST

The authors declare that they have no conflict of interest.

## AUTHOR CONTRIBUTIONS

**Yihu Zheng:** Conceptualization (lead); writing‐original draft (equal); data curation (supporting). **Qingqing Zhou:** Data curation (lead); methodology (equal); writing‐review and editing (lead). **Chang Zhao:** Methodology (equal); resources (equal); software (lead). **Junjian Li:** Investigation (lead); methodology (equal); resources (equal). **Zhengping Yu:** Resources (supporting); software (equal); writing‐original draft (equal). **Qiandong Zhu:** Conceptualization (supporting); data curation (lead); investigation (supporting).

## Data Availability

The data that support the finding of this study are available from the corresponding author upon reasonable request.
